# Developing and Validating a Nomogram Model for Predicting Ischemic Stroke Risk

**DOI:** 10.3390/jpm14070777

**Published:** 2024-07-22

**Authors:** Li Zhou, Youlin Wu, Jiani Wang, Haiyun Wu, Yongjun Tan, Xia Chen, Xiaosong Song, Yilin Wang, Qin Yang

**Affiliations:** 1 Department of Neurology, The First Affiliated Hospital of Chongqing Medical University, Chongqing 400016, China; 2 Department of Neurology, Chongzhou People’s Hospital, Chengdu 611200, China; 3 Department of Neurology, The Seventh People’s Hospital of Chongqing, Chongqing 400016, China; 4 Department of Neurology, The Ninth People’s Hospital of Chongqing, Chongqing 400016, China

**Keywords:** ischemic stroke, nomogram, predictors, LASSO

## Abstract

**Background and purpose**: Clinically, the ability to identify individuals at risk of ischemic stroke remains limited. This study aimed to develop a nomogram model for predicting the risk of acute ischemic stroke. **Methods**: In this study, we conducted a retrospective analysis on patients who visited the Department of Neurology, collecting important information including clinical records, demographic characteristics, and complete hematological tests. Participants were randomly divided into training and internal validation sets in a 7:3 ratio. Based on their diagnosis, patients were categorized as having or not having ischemic stroke (ischemic and non-ischemic stroke groups). Subsequently, in the training set, key predictive variables were identified through multivariate logistic regression and least absolute shrinkage and selection operator (LASSO) regression methods, and a nomogram model was constructed accordingly. The model was then evaluated on the internal validation set and an independent external validation set through area under the receiver operating characteristic curve (AUC-ROC) analysis, a Hosmer-Lemeshow goodness-of-fit test, and decision curve analysis (DCA) to verify its predictive efficacy and clinical applicability. **Results**: Eight predictors were identified: age, smoking status, hypertension, diabetes, atrial fibrillation, stroke history, white blood cell count, and vitamin B12 levels. Based on these factors, a nomogram with high predictive accuracy was constructed. The model demonstrated good predictive performance, with an AUC-ROC of 0.760 (95% confidence interval [CI]: 0.736–0.784). The AUC-ROC values for internal and external validation were 0.768 (95% CI: 0.732–0.804) and 0.732 (95% CI: 0.688–0.777), respectively, proving the model’s capability to predict the risk of ischemic stroke effectively. Calibration and DCA confirmed its clinical value. **Conclusions**: We constructed a nomogram based on eight variables, effectively quantifying the risk of ischemic stroke.

## 1. Introduction

Ischemic stroke is a leading cause of disability and death worldwide, particularly prevalent in developed countries, where it accounts for about 87% of all stroke cases. This condition imposes significant burdens on healthcare systems and economies [1,2]. The data from the 2019 Global Burden of Disease Study (GBD) indicate that stroke is the leading cause of disability-adjusted life years (DALYs) in China, imposing a significant burden on the country’s healthcare system [3]. Notably, factors such as population aging have led to a year-on-year increase in the incidence of ischemic stroke, which now accounts for 50.2% of total stroke mortality [3]. There are limited treatment options, such as tissue plasminogen activator (tPA) intravenous thrombolysis and intravascular mechanical thrombectomy, which are only used during the acute onset of ischemic stroke and are restricted by a narrow treatment window and severe complications [4,5]. Moreover, the implementation of successful secondary prevention and early rehabilitation treatment in primary care hospitals is not yet adequate [6]. Therefore, the early prevention and identification of high-risk individuals are particularly important, as are identifying new risk factors and optimizing risk assessment methods to accurately predict ischemic stroke [7]. This approach can not only enhance the timeliness and specificity of clinical interventions, thereby reducing the disease burden, but also deepen our understanding of ischemic stroke’s underlying mechanisms, promoting the development of new treatment strategies.

Current research on this topic is continually updating. Early studies have identified several risk factors for ischemic stroke: older age; female gender; genetic abnormalities (such as the APOE gene, MTHFR C677T, LPA gene variants, and the 9p21 locus) [8,9,10], history and family history of stroke; diabetes; hypertension; atrial fibrillation; chronic obstructive pulmonary disease; metabolic syndrome, etc. [11]. A study by He et al. constructed a predictive model for ischemic stroke based on logistic regression, but they only evaluated the model through a receiver operating characteristic (ROC) curve, without assigning and visualizing risk variables, which is inconvenient for clinical physicians [12]. Additionally, two pre-hospital stroke assessment nomograms have demonstrated that certain clinical characteristics can be used to assess the risk of ischemic stroke in emergency patients. The first nomogram incorporates sensory symptoms (pain), head and gaze deviation, unilateral arm/leg weakness or drift, visual field impairments, speech disturbances, asymmetric facial weakness, an age over 55, and blood glucose levels between 50 and 400 mg/dL [13]. The second nomogram focuses on demographic and historical data, including gender, history of diabetes, genetic background, history of coronary heart disease, smoking history, age, and a history of high blood pressure [14]. Although these tools are crucial in assessing the likelihood of an ischemic stroke and expediting the triage process, they have not yet undergone the necessary calibration, clinical evaluation, and validation to enhance their accuracy and practical applicability in real-world emergency settings. Moreover, these nomograms have not incorporated relevant laboratory data, which further limits their clinical application [13,14]. To date, a specific and practical prediction method is still lacking.

Nomograms, as intuitive visual prediction tools, have garnered increasing attention in the field of disease risk assessment due to their ease of understanding and convenience in clinical application [15]. The objective of this study is to conduct a comprehensive analysis of relevant clinical and laboratory indicators, evaluate key predictive factors, and develop and verify a nomogram model that is both simple and accurate for predicting the risk of ischemic stroke. This aims to provide clinicians with a concise and effective tool for risk assessment.

## 2. Materials and Methods

### 2.1. Study Population

This study was approved by the Medical Ethics Committee of the First Affiliated Hospital of Chongqing Medical University (Ethical Approval No.: 2022-115).

This clinical study was conducted at the First Affiliated Hospital of Chongqing Medical University and the People’s Hospital of Chongzhou City, Sichuan Province. The study was divided into two phases. The first phase (training set and internal validation set) involved a retrospective analysis of patients from the Department of Neurology at the First Affiliated Hospital of Chongqing Medical University, from October 2021 and October 2023. The second phase (external validation group) included patients from the Department of Neurology at Chongzhou People’s Hospital, from March 2022 to June 2023. All inpatients at the Department of Neurology were included in this clinical study. 

Participants who met the following inclusion criteria were enrolled in the study: (a) aged ≥18 years; (b) availability of all clinical and laboratory data; (c) possession of complete cranial magnetic resonance imaging (MRI) results; (d) informed consent obtained from the patient or their guardian. The exclusion criteria were as follows: (a) women who were pregnant or breastfeeding; (b) patients with a history of major surgery or trauma in the past 3 months; (c) patients with serious systemic internal diseases (severe anemia, severe malnutrition, severe liver and kidney failure, cachexia, etc.) [16]. The primary objective of this study is to establish a scoring system with high predictive accuracy to accurately differentiate between patients with ischemic and non-ischemic stroke.

Patients were divided into three groups (training set, internal validation set, and external validation set). The first group (training set) comprised 70% of the patients (n = 1493) from a retrospective study at the Department of Neurology of the First Affiliated Hospital of Chongqing Medical University, aimed at establishing a scoring system to differentiate between ischemic and non-ischemic stroke patients. The second group (internal validation set) included the remaining 30% of the patients (n = 641) from the First Affiliated Hospital of Chongqing Medical University to validate the diagnostic effectiveness of the scoring system. The third group (external validation group) included patients from Chongzhou People’s Hospital (n = 527) to further validate the prediction model. This study was conducted in accordance with the Declaration of Helsinki. All patients were required to provide written informed consent.

### 2.2. Data Collection

Data collection for this study was conducted using standardized clinical data collection forms administered by researchers to gather detailed information on the following aspects. All patient diagnoses were based on the International Classification of Diseases, Ninth Revision (ICD-9) or ICD-10 codes entered into the electronic medical record system at discharge, with repeated laboratory tests recording the first result after patient admission.

Initially, the clinical information and sociodemographic characteristics of hospitalized patients were extracted from the hospital’s electronic medical record system, encompassing general basic information such as age, gender, weight, height, and health risk behaviors. The Body Mass Index (BMI) of each participant was calculated as kg/m^2^. The personal information of the subjects was anonymized to ensure privacy protection. Subsequently, trained investigators collected the patients’ cranial MRI data and information regarding comorbidities, including hypertension, diabetes, dyslipidemia, cerebrovascular diseases, peripheral artery disease, depression, atrial fibrillation, coronary artery disease, chronic heart disease, thyroid disorders, chronic obstructive pulmonary disease, nephritis, nephrotic syndrome and kidney diseases, gastrointestinal diseases, malignancies, and surgical history (Table S1). Variables identified at the time of admission were recorded as pre-admission comorbidities. Laboratory test results were also collected, including complete blood count, hemoglobin (Hb), mean corpuscular volume (MCV), mean corpuscular hemoglobin concentration (MCHC), urea nitrogen (UN), creatinine (Crea), alanine aminotransferase (ALT), aspartate aminotransferase (AST), D-dimer (D2), fasting blood sugar (FBS), glycosylated hemoglobin (HbA1c), C-reactive protein (CRP), and low-density lipoprotein cholesterol (LDL), among other biochemical and clinical indicators. All laboratory evaluations were conducted in the laboratory departments of the First Affiliated Hospital of Chongqing Medical University and Chongzhou People’s Hospital. 

### 2.3. Statistical Analysis

Continuous variables are reported as means ± standard deviation (SD), medians, and interquartile range (IQRs), while categorical variables are reported as percentages. Differences between the ischemic stroke group and the non-ischemic stroke group were compared using the *t*-test or Mann-Whitney U test. For categorical variables, comparisons were made using Pearson’s χ^2^ test and Fisher’s exact test. All *p*-values were two-sided, and a *p*-value of less than 0.05 was considered statistically significant. 

In the training set data, “occurrence of ischemic stroke” was used as the outcome variable. Binary logistic regression analysis was applied to identify independent risk factors for ischemic stroke. Additionally, LASSO (least absolute shrinkage and selection operator) regression was used within the training set to select the optimal predictors by choosing the best lambda (λ) value. Only variables that were significant in both multivariate logistic regression and LASSO regression were chosen as the final selection variables for constructing the nomogram model.

The predictive performance of the model was evaluated using the training set, internal validation set, and external validation set. Evaluation metrics included the discriminative ability of the nomogram in the training and validation sets, measured by the area under the receiver operating characteristic curve (AUC-ROC) curve. The stability and prediction accuracy of the model were validated through bootstrap resampling, with 1000 repeats to ensure reliability. Furthermore, calibration curves and clinical decision curves were also used to assess the model’s predictive precision and clinical utility. Calibration curves evaluate the agreement between observed outcomes and predictions, while clinical decision curves analyze the clinical benefits of using the nomogram across different threshold probabilities, aiding in decision-making processes. 

In this study, R software (Version 4.3.2) was employed for statistical analysis and model construction. The glmnet package was utilized for performing LASSO regression, which is effective for variable selection and regularization in predictive models. The rms package was used to build logistic regression models and construct nomograms, which are graphical representations of the regression model that allow for the prediction of outcomes based on individual patient characteristics. ROC curve analysis, which is essential for assessing the diagnostic ability of the model, was conducted using the pROC package. Calibration curves, which compare the predicted probabilities of outcomes with the observed outcomes, were analyzed using available functions in the Calibration Curves package. Decision curve analysis (DCA), which evaluates the clinical utility of predictive models, was performed with the rmda package. All statistical tests were two-sided, and a *p*-value of less than 0.05 was considered statistically significant. This comprehensive approach ensured a robust evaluation of the model’s performance and its applicability in clinical settings.

## 3. Results

### 3.1. Study Populations

**Figure 1:**
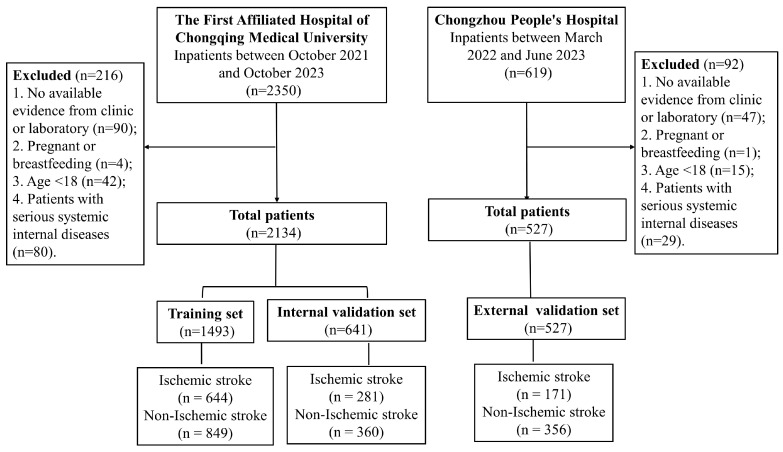
Flowchart of participant selection.

Table 1 summarizes the basic characteristics of the training set, internal validation set, and external validation set in this study. A total of 2134 patients from the Department of Neurology at the First Affiliated Hospital of Chongqing Medical University were included in the study, with 70% randomly assigned to the training group (n = 1493) and the remaining 30% assigned to the internal validation group (n = 641). Additionally, 527 patients from Chongzhou People’s Hospital were included in the external validation set. A detailed patient selection flowchart is shown in Figure 1.

### 3.2. Multivariate Logistic Regression Analysis of Independent Risk Factors for Ischemic Stroke

As indicated in Table 2, univariate analysis identified 21 variables significantly associated with ischemic stroke, all of which were selected as potential predictors for multivariate logistic regression. The results of the multivariate analysis showed that age, smoking, diabetes diagnosis, hypertension diagnosis, previous strokes (transient ischemic attack and ischemic stroke), atrial fibrillation, white blood cell count, and vitamin B12 levels are independent risk factors for ischemic stroke.

### 3.3. Construction of a Nomogram in the Training Set

**Figure 2:**
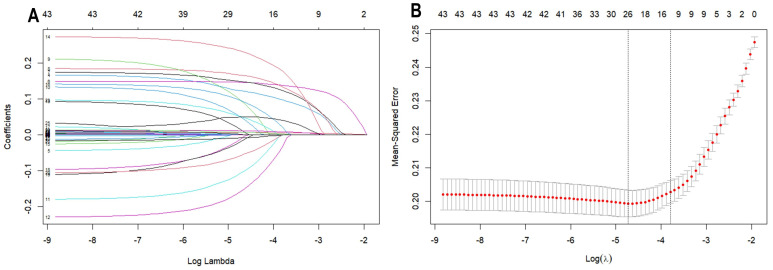
LASSO regression analysis with tenfold cross-validation of predictors of acute ischemic stroke. (**A**) This is a coefficient profile plot created based on the log(λ) sequence. The x-axis represents the logarithm of λ, while the y-axis represents the regression coefficients. Each colored solid line in the graph represents a variable. As log(λ) increases, the coefficients of the variables continuously decrease, with some variable coefficients approaching zero. (**B**) A 10-fold cross-validation curve for LASSO regression. The x-axis represents the logarithm of λ, and the y-axis represents the mean squared error (MSE). The dashed line on the left side of the graph indicates the λ value (0.01606023) corresponding to the minimum MSE, while the dashed line on the right side indicates the λ value (0.02245227) that is one standard deviation away from the minimum MSE. In this study, the selection of predictors is based on the λ value that is one standard deviation away from the minimum MSE (the right dashed line), where 8 non-zero coefficients were selected.

Using LASSO regression analysis to screen clinical and laboratory characteristics, a total of 12 independent variables were found to be associated with ischemic stroke (*p* < 0.05). These variables include age, smoking, hypertension diagnosis, diabetes diagnosis, previous strokes (transient ischemic attack and ischemic stroke), depression, atrial fibrillation, cardiovascular disease, white blood cell count, red blood cell count, vitamin B12 levels, and fasting blood glucose levels (as shown in Figure 2).

**Figure 3:**
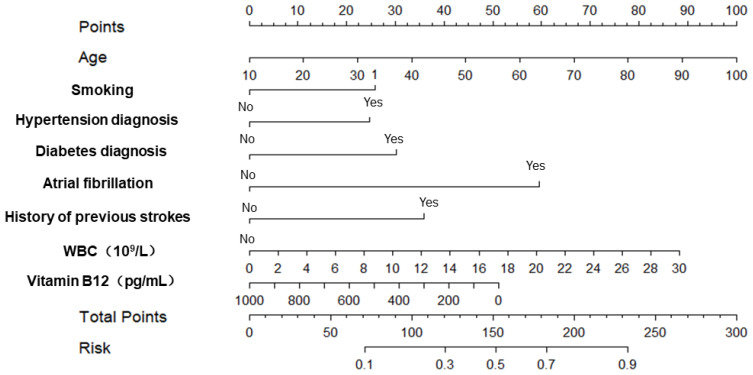
The nomogram for predicting the risk of acute ischemic stroke.

Therefore, based on the results of the multivariate logistic regression analysis and LASSO regression analysis, we selected age, smoking, hypertension diagnosis, diabetes diagnosis, atrial fibrillation, history of previous strokes (transient ischemic attack and ischemic stroke), white blood cell count, and vitamin B12 levels as predictors to construct a nomogram (as shown in Figure 3).

### 3.4. Diagnostic Performance of the Scoring System in the Training Set and Validation Set

**Figure 4:**
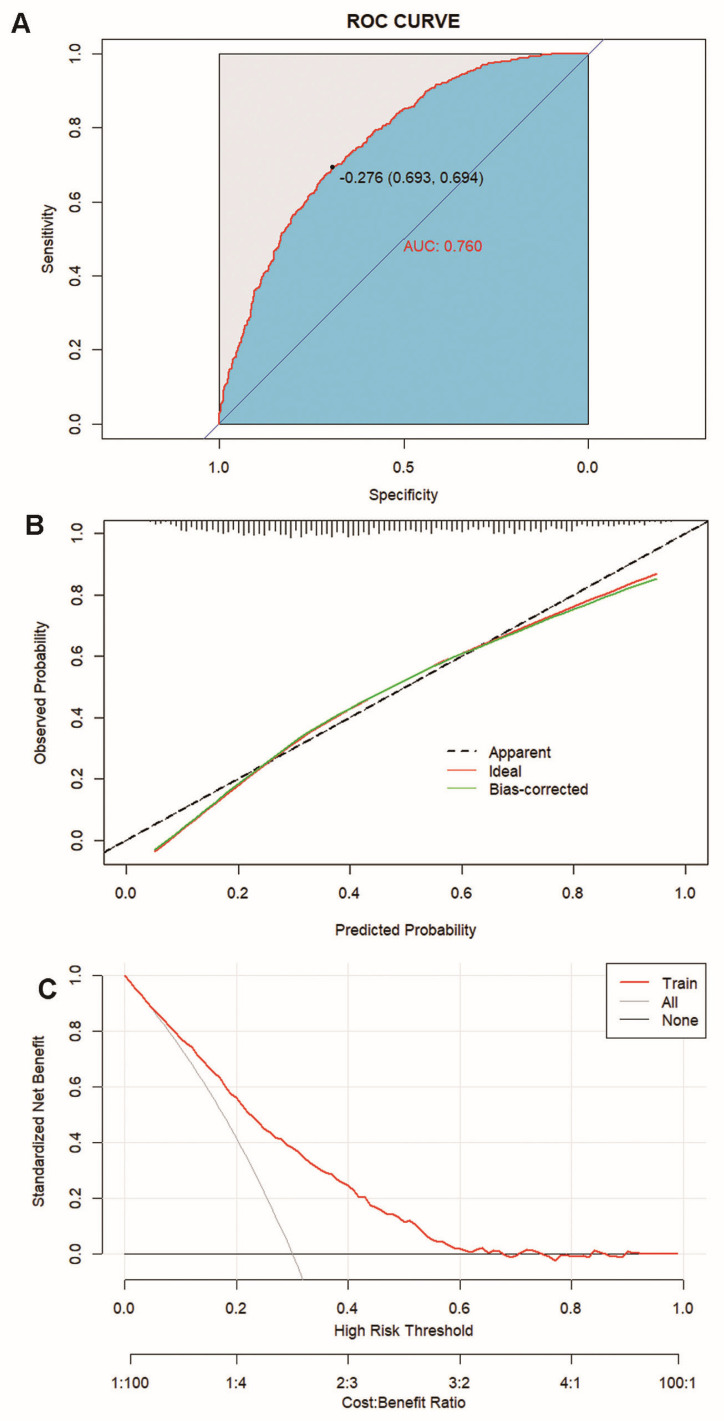
Calibration and clinical use of a diagnostic nomogram for the discrimination of ischemic stroke and non-ischemic stroke. (**A**) AUC-ROC for identifying the nomogram. (**B**) Calibration curve of the diagnostic nomogram. (**C**) DCA of the diagnostic nomogram.

Figure 4 illustrates that the nomogram demonstrates good discriminative ability in the training set, with an AUC-ROC of 0.760 (95% CI: 0.736–0.784) (Figure 4A), and the calibration curve indicates that the nomogram is well calibrated in the training set (Figure 4B). Additionally, the DCA shows that in the training set, a threshold probability ranging from 5% to 62% using this nomogram for predicting ischemic stroke could yield a greater net benefit.

**Figure 5:**
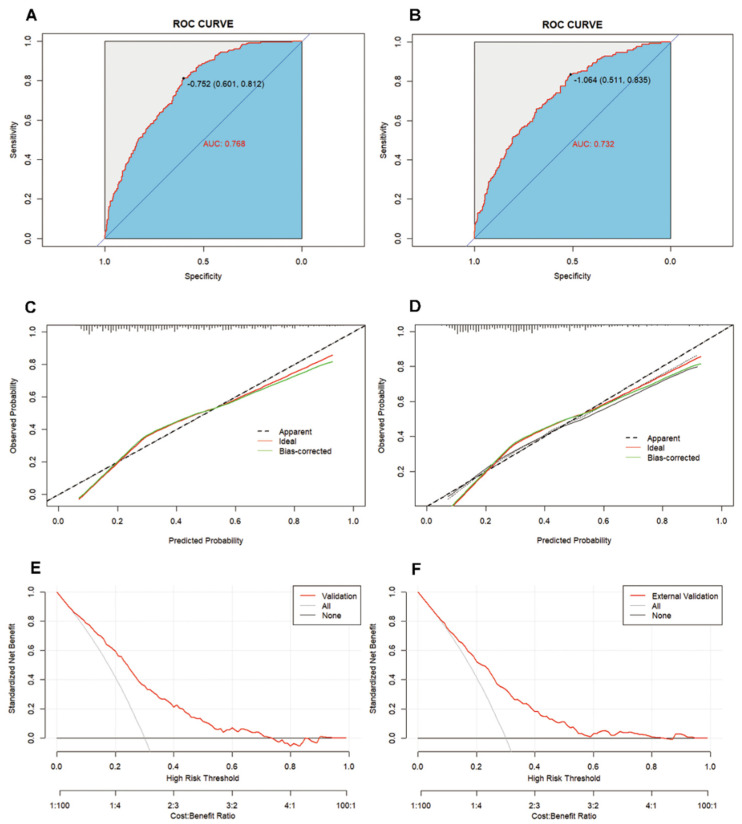
Discrimination and calibration of the scoring system for the discrimination of ischemic stroke and non-ischemic stroke. ROC curves of the nomogram in the internal validation set (**A**) and external validation set (**B**). Calibration curves of the nomogram in the internal validation set (**C**) and external validation set (**D**). DCA of the nomogram internal validation set (**E**) and external validation set (**F**).

Figure 5 demonstrates that by applying the AUC-ROC score on both the internal and external validation sets, the nomogram is confirmed to possess excellent discriminative power in predicting the risk of ischemic stroke. The internal validation set yielded an AUC-ROC value of 0.768 (95% CI: 0.732–0.804) (Figure 5A), while the external validation set had a value of 0.732 (95% CI: 0.688–0.777) (Figure 5B), both of which show a high degree of consistency, further emphasizing the reliability of the nomogram in determining the risk of ischemic stroke. Additionally, the calibration curves validate that the scoring system displays precise calibration in both the internal validation set (Figure 5C) and external validation set (Figure 5D), suggesting no significant difference between the predicted probabilities and the actual occurrences. Crucially, the DCA for the internal validation set (Figure 5E) reveals that within a threshold probability range of 5–72%, utilizing this nomogram can yield a greater net benefit; similarly, the DCA for the external validation set (Figure 5F) indicates that within a threshold probability range of 7–81%, an increased net benefit can also be observed.

## 4. Discussion

Facing the challenges of an aging population and the increasing burden of stroke, developing and validating more effective treatment and prevention strategies for ischemic stroke have become especially important. This study, through an analysis of data collected from neurology inpatients, identified eight risk factors closely associated with the occurrence of ischemic stroke. These factors include age, smoking, hypertension diagnosis, diabetes diagnosis, atrial fibrillation, history of previous strokes (including transient ischemic attacks and ischemic stroke), white blood cell count, and vitamin B12 levels. Based on these eight variables, we developed and validated a nomogram that demonstrates good discriminative ability, calibration, and clinical utility. This nomogram can predict the probability of ischemic stroke occurrence, providing a basis for the individualized identification of ischemic stroke risk.

However, in existing research, the majority of studies focus on identifying indicators or predictive models that affect post-stroke recovery, with only a few studies establishing models for the early recognition of ischemic stroke. For example, one study included a consecutive series of 112 patients with ischemic cerebrovascular disease and developed a nomogram using a combination of clinical and imaging methods. Although the DCA indicated that this model could generate better net clinical benefits when the threshold probability was between 0.16 and 0.82, its clinical utility was limited due to the small sample size and the high costs of imaging equipment, as well as the financial burden on patients [17]. Another study comparing 2151 middle-aged stroke patients (66.9 ± 11.9 years) with 1527 healthy controls (59.6 ± 13.9) found that non-invasive factors including gender, history of diabetes, genetic history, history of coronary heart disease, smoking history, and age could identify high-risk groups for ischemic stroke. However, the lack of model calibration checks and clinical decision curve assessments still leaves questions about the preciseness of its application [14].

In our study, through multivariate regression analysis and LASSO regression, we identified eight independent risk factors, consistent with the existing literature. Firstly, advanced age and smoking were recognized as independent risk factors for ischemic stroke, aligning with the literature [3]. With aging, the human body undergoes various vascular changes, including arterial stiffening, reduced blood flow, and narrowing of the vascular lumen, all of which elevate the risk of cerebral vascular blockage leading to ischemic stroke [18]. Our statistical data indicate that the incidence rate of ischemic stroke is particularly high among the elderly, highlighting the importance of focusing on the health status and lifestyle modifications of this population in the prevention and management of ischemic stroke. Smoking is a primary risk factor for initial stroke events [19]. Mendelian research has demonstrated that smoking increases the risk of ischemic stroke, with an odds ratio (OR) of 1.22 (95% CI = 1.12–1.34), and the risk escalates with the duration of smoking [20]. Cigarette smoke contains peroxides (such as nitrogen oxides and free radicals) and other toxic substances that may increase stroke risk through endothelial dysfunction, lipid oxidation, inflammation, platelet activation, thrombogenesis, and enhanced coagulability. Furthermore, nicotine may reduce cerebral blood flow [21].

The independent association of hypertension once again underscores the importance of blood pressure control in the prevention of ischemic stroke. Currently, hypertension is recognized as the most significant risk factor affecting the occurrence and recurrence of ischemic stroke [22]. The mechanism may be related to cerebrovascular remodeling caused by the reduction in the cerebral vascular lumen diameter and the increase in vessel wall thickness that occur with hypertension [23,24]. Research has found a positive correlation between systolic blood pressure and the occurrence of ischemic stroke. Particularly in some Asian countries, ongoing hypertension treatment has been considered a core strategy for stroke prevention [25]. Similarly, in diabetes, abnormal glucose states can disrupt normal endothelial function through oxidative stress, the activation of protein kinase C, and receptors for advanced glycation end products, thereby accelerating arteriosclerosis and promoting the formation of atherosclerotic plaques in the carotid arteries [26,27]. Impaired fasting glucose is also associated with endothelial dysfunction and intima-media thickening, which are linked to an increased risk of stroke [28]. Furthermore, the 3–5 thetiareregistry.org project indicates that patients who have experienced a transient ischemic attack or mild stroke have a risk of about 5% for stroke in the following year [29]. More importantly, patients with ischemic stroke who also have atrial fibrillation suffer from more disabilities and a higher degree of initial severity compared to those without atrial fibrillation, and the duration of atrial fibrillation has a direct and significant association with ischemic stroke [30,31].

WBC is a critical marker of inflammation in the body, and its role as an independent risk factor for ischemic stroke has been confirmed. Previous studies have shown that inflammation is closely associated with all stages of ischemic stroke, not only facilitating the formation of ischemic damage but also exacerbating the deterioration of neurological function [32,33]. Research indicates that even in healthy populations, higher white blood cell counts are closely linked to an increased risk of ischemic stroke [34]. Another study revealed that elevated total white blood cell and neutrophil counts upon admission are significantly associated with the presence of large-artery occlusive stroke and possess a moderate ability to differentiate this type of stroke [35].

In this study, the negative correlation between vitamin B12 levels and stroke risk is a unique finding. Vitamin B12 not only plays a role by regulating homocysteine levels, but also acts as a superoxide scavenger, with direct antioxidative, antithrombotic, and endothelial protective functions, thereby participating in the prevention of arteriosclerosis formation [36]. Previous literature reviews have already revealed multiple biological and clinical associations between vitamin B12 and ischemic stroke [37,38]. Furthermore, vitamin B12 levels are negatively correlated with the degree of arteriosclerosis and closely associated with the occurrence of cerebral infarction [39,40]. Studies have indicated that higher levels of vitamin B12 are related to a lower risk of ischemic stroke [41]. These findings, in conjunction with our research, suggest that vitamin B12 may offer a protective effect for patients with ischemic stroke.

Through the constructed nomogram, we provide a quantitative tool that integrates multiple risk factors to predict an individual’s risk of ischemic stroke, aiding in clinical decision-making. The nomogram’s AUC-ROC values are 0.768 and 0.732 for the internal and external validation sets, respectively, demonstrating good predictive efficiency. Additionally, the DCA curve indicates that the nomogram offers a wide range of threshold probabilities in predicting ischemic stroke, contributing to the early identification of ischemic stroke and the optimization of care protocols. 

However, this study is not without limitations. Firstly, all included patients come from a single center, which may introduce selection bias. Secondly, as this is a cross-sectional study, definitive conclusions may not be drawn. Importantly, the COVID-19 pandemic period overlapped with our study period, which could have influenced patient profiles and outcomes, an aspect not specifically accounted for in our analysis. Lastly, some important potential risk factors, such as exercise habits and dietary patterns, were not included in the analysis, which could affect our results. Future research should expand the sample source and include more variables to validate our findings and further optimize the nomogram.

It is worth mentioning that although the nomogram demonstrated good predictive capability statistically, its effectiveness in practical application needs further validation through prospective studies. We acknowledge that vitamin B12 levels may not be part of routine clinical activity in all medical settings, which could limit the nomogram’s applicability. While the inclusion of vitamin B12 offers additional predictive value, the practical application of our nomogram might be limited to healthcare settings where such testing is standard, or it may prompt a reevaluation of routine testing protocols in light of evolving evidence. Therefore, we encourage other research centers to independently validate the nomogram and make necessary adjustments according to the characteristics of different populations. Moreover, future studies should consider incorporating more commonly measured clinical variables and conducting randomized controlled trials to evaluate whether interventions guided by the nomogram can improve clinical outcomes for patients. 

## 5. Conclusions

In summary, our study introduces a novel and practical nomogram, which utilizes age, smoking status, diagnoses of hypertension and diabetes, atrial fibrillation, history of previous strokes (including transient ischemic attacks, ischemic stroke, and intracerebral hemorrhage), white blood cell count, and vitamin B12 levels, for predicting the likelihood of ischemic stroke in patients. This nomogram has been internally validated and proven to be a valuable tool for the risk assessment and formulation of prevention strategies for ischemic stroke, thereby effectively aiding clinicians in medical decision-making. Nonetheless, further research is warranted to confirm the nomogram’s effectiveness.

## Figures and Tables

**Table 1:** Baseline characteristics of subjects in the training set and validation set.

Variables	Total (n = 2134)	Training Set (n = 1493)	Internal Validation Set (n = 641)	External Validation Set (n = 527)
Age (years)	63 (51, 72)	63 (51, 72)	62 (51, 72)	68 (57, 75)
BMI (kg/m^2^)	23.03 (21.09, 25.39)	23.13 (21.11, 25.63)	22.925 (20.96, 25.09)	23.44 (21.10, 25.51)
Ischemic stroke (%)	925 (43.37)	644 (43.15)	281 (43.89)	171 (32.44)
Female (%)	1044 (48.92)	730 (48.92)	326 (50.78)	244 (46.30)
Smoking (%)	834 (39.09)	590 (39.52)	244 (38.09)	144 (27.32)
Alcohol drinking (%)	633 (29.69)	438 (29.37)	195 (30.41)	115 (21.82)
Hypertension diagnosis (%)	847 (39.70)	604 (40.46)	243 (37.93)	266 (50.47)
Diabetes diagnosis (%)	412 (19.33)	290 (19.42)	123 (19.12)	105 (19.92)
Previous ischemic stroke or transient ischemic attacks (%)	150 (7.06)	114 (7.66)	36 (5.64)	22 (4.17)
Carotid artery disease (%)	7 (0.33)	6 (0.40)	1 (0.16)	36 (6.83)
Peripheral arterial disease (%)	12 (0.56)	8 (0.54)	4 (0.63)	11 (2.09)
Intracerebral hemorrhage (%)	33 (1.55)	25 (1.68)	8 (0.01)	1 (0.19)
Depression (%)	16 (0.74)	11 (0.74)	3 (0.47)	5 (0.95)
Lipid-related diagnosis (%)	25 (1.18)	17 (1.14)	8 (1.25)	14 (2.66)
Atrial fibrillation (%)	50 (2.35)	30 (2.02)	20 (3.13)	14 (2.66)
Coronary heart disease (%)	143 (6.68)	103 (6.92)	39 (6.11)	7 (1.33)
Chronic heart disease (%)	47 (2.21)	32 (2.15)	15 (2.35)	10 (1.90)
Thyroid disease (%)	23 (1.08)	16 (1.08)	7 (1.10)	1 (0.19)
Chronic obstructive pulmonary disease (%)	25 (1.18)	19 (1.28)	6 (0.94)	12 (2.28)
Nephritis, nephrotic syndrome, and nephrosis (%)	30 (1.41)	18 (1.21)	12 (1.88)	7 (1.33)
Gastrointestinal disorders (%)	113 (5.32)	80 (5.38)	33 (5.17)	NA^#^
Malignant neoplasms (%)	41 (1.93)	31 (2.08)	10 (1.57)	NA^#^
Surgery (%)	129 (6.07)	86 (5.78)	43 (6.74)	NA^#^
Family history	105 (4.94)	76 (5.11)	29 (4.55)	NA^#^
Hematological findings				
WBC (10^9^/L)	6.99 (5.68, 8.87)	7.06 (5.70, 8.89)	6.84 (5.65, 8.78)	6.00 (4.95, 7.25)
RBC (10^12^/L)	4.49 (4.11, 4.87)	4.50 (4.12, 4.87)	4.47 (4.09, 4.86)	4.25 (3.85, 4.69)
PLT (10^9^/L)	206 (165, 251)	206 (165, 251)	207.50 (165.00, 250.75)	154.50 (118.25, 199.75)
Hb (g/L)	136.00 (125.00, 148.00)	137.00 (125.00, 148.00)	135.00 (125.00, 149.00)	130.00 (119.00, 142.75)
MCV (fl)	92.40 (89.5, 95.4)	92.40 (89.50, 95.40)	92.70 (89.40, 95.58)	94.00 (91.00, 97.48)
MCHC (g/L)	330 (323, 338)	331 (323, 338)	330 (322.00, 338.00)	326.00 (321.00, 332.00)
HCT (%)	41.35 (38.0, 44.50)	41.40 (38.10, 44.40)	41.20 (37.90, 44.68)	39.85 (36.40, 43.40)
BUN (mmol/L)	5.60 (4.525, 6.90)	5.50 (4.50, 6.90)	5.60 (4.60, 6.88)	5.42 (4.42, 6.78)
Crea (μmol/L)	66.00 (56.00, 80.00)	66.00 (56.00, 80.00)	65.00 (55.00, 81.00)	70.30 (60.50, 83.18)
ALT (U/L)	26 (20, 35)	26 (20, 35)	26 (20, 35)	20 (15, 31)
AST (U/L)	25 (21, 31)	25 (21, 31)	26 (21, 32)	23 (19, 30)
FBG (mmol/L)	5.40 (4.90, 6.30)	5.40 (4.90, 6.30)	5.40 (4.90, 6.30)	5.40 (4.87, 6.50)
HbA1c (%)	5.70 (5.40, 6.10)	5.70 (5.40, 6.10)	5.70 (5.40, 6.10)	5.60 (5.30, 6.20)
CRP (mg/mL)	1.13 (0.49, 4.10)	1.15 (0.48, 3.95)	1.11 (0.53, 4.28)	1.96 (0.75, 4.95)
PCT (ng/mL)	0.03 (0.02, 0.05)	0.03 (0.02, 0.05)	0.03 (0.02, 0.05)	0.11 (0.06, 0.21)
LDL (mmol/L)	2.50 (1.94, 3.12)	2.51 (1.94, 3.14)	2.50 (1.93, 3.06)	2.32 (1.77, 2.82)
D2 (mg/L FEU)	0.37 (0.21, 0.83)	0.37 (0.21, 0.84)	0.37 (0.21, 0.82)	0.39 (0.24, 0.72)
Vitamin B12 (pg/mL)	281.00 (205.00, 396.00)	277.50 (203.37, 393.00)	287.00 (211.25, 403.75)	449.00 (322.00, 588.00)
Folate (ng/mL)	9.50 (6.60, 14.90)	9.30 (6.50, 14.725)	10.25 (7.0, 15.60)	8.75 (6.16, 12.33)
tHcy (μmol/L)	12.60 (10.00, 16.10)	12.60 (10.00, 16.43)	12.50 (9.90, 15.78)	9.90 (8.00, 13.20)

Data are presented as medians (IQR), numbers, or percentages. ^#^NA indicates that the data for these items were not collected in the external validation set. BMI = body mass index; WBC = white blood cell; RBC = red blood count; PLT = platelets; Hb = hemoglobin; MCV = mean corpuscular volume; MCHC = mean corpuscular hemoglobin concentration; HCT = hematocrit; tHcy = total homocysteine; BUN = blood urea nitrogen; Crea = creatinine; ALT = alanine transaminase; AST = aspartate transaminase; D2 = D-dimer; FBG = fasting blood glucose; HbA1c = glycosylated hemoglobin; CRP = C-reactive protein; PCT = procalcitonin; LDL = low-density lipoprotein.

**Table 2:** Univariate and multivariate logistic regression analysis of cases and controls in the training set.

Variables	Univariate Analysis		Multivariate Analysis	
	OR (95% CI)	*p*-Value	OR (95% CI)	*p*-Value
Age (years)	1.041 (1.033, 1.049)	<0.001	1.0349 (1.026, 1.044)	<0.001
BMI (kg/m^2^)	1.043 (1.011, 1.076)	0.008	NA	0.532796
Smoking	1.979 (1.603, 2.446)	<0.001	2.124 (1.567, 2.891)	<0.001
Alcohol drinking	1.441 (1.053, 1.801)	0.001	NA	0.302699
Diabetes diagnosis	3.141 (2.396, 4.142)	<0.001	2.257 (1.646, 3.109)	<0.001
Hypertension diagnosis	3.383 (2.725, 4.207)	<0.001	1.945 (1.508, 2.509)	<0.001
Previous ischemic stroke or transient ischemic attacks	4.339 (2.776, 7.015)	<0.001	2.599 (1.588, 4.373)	<0.001
Atrial fibrillation (%)	6.620 (2.946, 17.691)	<0.001	4.733 (1.946, 13.458)	0.002
Coronary heart disease	1.574 (1.054, 2.363)	0.027	NA	0.638723
Chronic heart disease	2.320 (1.191, 4.726)	0.016	NA	0.057050
Family history	1.595 (1.007, 2.546)	0.048	NA	0.117320
WBC (10^9^/L)	1.096 (1.056, 1.139)	<0.001	1.062 (1.015, 1.111)	0.009
RBC (10^12^/L)	1.355 (1.145, 1.608)	<0.001	NA	0.056671
Hb (g/L)	1.009 (1.003, 1.015)	0.002	NA	0.775278
HCT (%)	1.030 (1.010, 1.052)	0.003	NA	0.690021
Crea (μmol/L)	1.003 (1.001, 1.006)	0.024	NA	0.288911
FBG (mmol/L)	1.091 (1.039, 1.148)	0.001	NA	0.052744
D2 (mg/L FEU)	1.084 (1.015, 1.161)	0.018	NA	0.550925
Vitamin B12 (pg/mL)	0.998 (0.997, 0.999)	<0.001	0.998 (0.998, 0.999)	<0.001
HbA1c (%)	1.154 (1.053, 1.267)	0.002	NA	0.384580
tHcy (μmol/L)	1.001 (0.992, 1.011)	0.076	NA	0.344208

“NA” indicates that in multivariate analysis, the statistical data for some variables are no longer statistically significant after considering the effects of other variables, and therefore, they are not included in the final model. OR = odds ratio; CI = confidence interval; WBC = white blood cell; RBC = red blood count; Hb = hemoglobin; HCT = hematocrit; Crea = creatinine; D2 = D-dimer; FBG = fasting blood glucose; HbA1c = glycosylated hemoglobin; tHcy = total homocysteine.

## Data Availability

The data not published within this article are available from the corresponding author on reasonable request.

## Supplementary Materials

The following supporting information can be downloaded at: https://www.mdpi.com/article/10.3390/jpm14070777/s1, Table S1: Diagnostic criteria of covariates.

## Abbreviations

LASSO: least absolute shrinkage and selection operator; ROC: receiver operating characteristic; AUC: area under the curve; CI: confidence interval; DCA: decision curve analysis; GBD: Global Burden of Disease Study; DALYs: disability-adjusted life years; tPA: tissue plasminogen activator; MRI: magnetic resonance imaging; BMI: body mass index; Hb: hemoglobin; MCV: mean corpuscular volume; MCHC: mean corpuscular hemoglobin concentration; UN: urea nitrogen; Crea: creatinine; ALT: alanine aminotransferase; AST: aspartate aminotransferase; D2: D-dimer; FBS: fasting blood sugar; HbA1c: glycosylated hemoglobin; CRP: C-reactive protein; LDL: low-density lipoprotein cholesterol; SD: standard deviation; IQR: interquartile range; WBC: white blood cell; RBC: red blood count; PLT: platelets; HCT: hematocrit; tHcy: total homocysteine; BUN: blood urea nitrogen; FBG: fasting blood glucose; PCT: procalcitonin; OR: odds ratio; MSE: mean squared error.
