# Supplementary material for: Developing and Validating a Nomogram Model for Predicting Ischemic Stroke Risk
Source: J Pers Med. 2024 Jul 22;14(7):777. doi: 10.3390/jpm14070777 (PMC11277803; doi:10.3390/jpm14070777)
Supplement: Supplementary file 1 [file jpm-14-00777-s001.zip › jpm-3079739-supplementary.pdf]

Table S1. Diagnostic criteria of covariates

Variable	Diagnostic criteria
Diabetes	The diagnostic criteria for diabetes are: the doctor told you have diabetes or glycohemoglobin HbA1c (%) greater than 6.5 or use of diabetes medication or insulin. This variable was subdivided into two groups: yes and no.
Hypertension	The diagnostic criteria for hypertension are: the doctor told you have hypertension or use of medication for hypertension or systolic more than 140 or diastolic more than 90. Average blood pressure was calculated by the following protocol: The diastolic reading with zero is not used to calculate the diastolic average; If all diastolic readings were zero, then the average would be zero; If only one blood pressure reading was obtained, that reading is the average; If there is more than one blood pressure reading, the first reading is always excluded from the average. This variable was subdivided into two groups: yes and no.
Dyslipidemia	Hyperlipidemia refers to an elevation of triglycerides or cholesterol in the blood, or the use of lipid-lowering drugs. Hypertriglyceridemia is defined as triglycerides levels greater than or equal to 1.7 mmol/L, while hypercholesterolemia is defined as total cholesterol levels greater than or equal to 5.2 mmol/L, or low-density lipoprotein levels greater than or equal to 3.37mmol/L. This variable was subdivided into two groups: yes and no.
Cerebrovascular diseases	The diagnostic criteria for cerebrovascular diseases are: the doctor informed you that you have cerebrovascular disease or it was confirmed through medical examination. This variable was subdivided into two groups: yes and no.
Peripheral artery disease	The diagnostic criteria for peripheral artery disease are: the doctor informed you that you have peripheral artery disease or it was confirmed through medical examination. This variable was subdivided into two groups: yes and no.
Depression	The diagnostic criteria for depression are: the doctor informed you that you have depression, or you are using antidepressant medication, or it was confirmed through standardized mental health assessment tools (e.g., a PHQ-9 score of 10 or higher). This variable was subdivided into two groups: yes and no.
Atrial fibrillation	The diagnostic criteria for atrial fibrillation are: the doctor informed you that you have atrial fibrillation or it was confirmed through an electrocardiogram (ECG/EKG). This variable was subdivided into two groups: yes and no.
Coronary artery disease	The diagnostic criteria for coronary artery disease are: the doctor informed you that you have coronary artery disease or it was confirmed through coronary angiography, cardiac CT, or magnetic

	resonance imaging (MRI). This variable was subdivided into two groups: yes and no.
Chronic heart disease	The diagnostic criteria for chronic heart disease are: the doctor informed you that you have chronic heart disease or it was confirmed through relevant medical examinations such as echocardiography, electrocardiogram (ECG/EKG), or magnetic resonance imaging (MRI). This variable was subdivided into two groups: yes and no.
Thyroid disorders	The diagnostic criteria for thyroid disorders are: the doctor informed you that you have a thyroid disorder or you are using related medication, or it was confirmed through blood tests (e.g., TSH, T3, T4 levels) or imaging studies. This variable was subdivided into two groups: yes and no.
Chronic obstructive pulmonary disease	COPD including chronic bronchitis and/or emphysema were defined according to the self-reported diagnosis. This variable was subdivided into two groups: yes and no.
Nephritis	The diagnostic criteria for nephritis are: the doctor informed you that you have nephritis or it was confirmed through urinalysis (e.g., proteinuria, hematuria) and blood tests (e.g., serum creatinine, blood urea nitrogen levels) or imaging studies (e.g., renal ultrasound, CT). This variable was subdivided into two groups: yes and no.
Nephrotic syndrome and kidney diseases	CKD is defined as an eGFR of less than 60 mL/min/1.73 m^2. Renal function was calculated as an estimated glomerularfiltration rate (eGFR) with the formula developed by theChronic Kidney Disease Epidemiology Collaboration (CKD-EPI). This variable was subdivided into two groups: yes and no.
Gastrointestinal diseases	The diagnostic criteria for gastrointestinal diseases are: the doctor informed you that you have a gastrointestinal disease or you are using related medication, or it was confirmed through endoscopic examinations (e.g., gastroscopy, colonoscopy), imaging studies (e.g., abdominal ultrasound, CT, MRI), or laboratory tests (e.g., fecal occult blood test). This variable was subdivided into two groups: yes and no.
Malignancies	The diagnostic criteria for malignancies are: the doctor informed you that you have a malignancy or you are undergoing related treatment, or it was confirmed through histopathological examination, imaging studies (e.g., CT, MRI, PET-CT), or tumor marker tests. This variable was subdivided into two groups: yes and no.
Surgical history	The diagnostic criteria for surgical history are: the doctor informed you that you have undergone surgery or it was confirmed through medical records. This variable was subdivided into two groups: yes and no.
